# Application of adenosine stress echocardiography in the prognosis of acute myocardial infarction following percutaneous coronary interventional therapy

**DOI:** 10.3892/etm.2013.1193

**Published:** 2013-07-01

**Authors:** LIHUI REN, YONG LIU, JING LIN, HUIMING YE, PING WANG, YINGPING LIU

**Affiliations:** Department of Cardiology, Beijing Shijitan Hospital Affiliated to Capital Medical University, Beijing 100038, P.R. China

**Keywords:** acute myocardial infarction, adenosine stress echocardiography, prognosis, coronary artery disease

## Abstract

The aim of this study was to investigate the application of adenosine stress echocardiography (ASE) in the prognosis of acute myocardial infarction (AMI) following percutaneous coronary intervention (PCI). A total of 79 patients with AMI who underwent PCI were selected for the study. ASE testing was performed within one week following the PCI. Subsequent to the ASE, the patients with ≥5% increases in the left ventricular ejection fraction (LVEF) levels were included in the improved LVEF group, while patients with <5% increases in LVEF levels were included in the unimproved LVEF group. A follow-up study was performed during the 24 months subsequent to the ASE. The incidence of major adverse cardiovascular events (MACEs) was observed and compared between the two groups and logistic regression analysis was applied to identify the risk factors for clinical prognosis. There were no significant differences in Killip classification, LVEF, left ventricular end-diastolic diameter or blood plasma B-type natriuretic peptide concentration between the two groups following PCI. The incidence of MACEs in the improved LVEF group was significantly lower than that in unimproved LVEF group (14.29 versus 43.24%, respectively; P<0.05). Logistic regression analysis identified LVEF increases of <5% and segment improvements of ≤3 as the risk factors for the clinical prognosis of AMI following treatment with PCI. Therefore, ASE is an effective method of assessing the clinical effect of PCI treatment, which may be utilized to predict the incidence of MACEs following PCI.

## Introduction

Coronary artery disease is a frequently occurring disease, with an incidence that significantly increases annually. The incidence of coronary artery disease has been reported to have increased to 67% between 1993 and 2007 in China, with an average annual increase of 1.7% (1). Acute myocardial infarction (AMI) is a serious cardiovascular disease with high morbidity and mortality. The most effective method of saving the endangered necrotic myocardium is the early and effective opening of the culprit artery.

Following recanalization, the impaired myocardial function may be fully restored in certain patients, and the prognosis of these patients is often improved (2). However, a number of patients continue to experience gradual ventricular wall thinning and ventricular enlargement. In addition, left ventricular (LV) remodeling has been identified to result in a poor prognosis (3). Therefore, predicting the prognosis of patients by identifying coronary revascularization is of particular significance for the treatment of coronary heart disease, with implications for program formulation, efficacy evaluation, the degrees of risk stratification and prevention of adverse cardiovascular events.

Adenosine is a heterocyclic molecule with a molecular weight of 267.25. Adenosine is synthesized through a metabolic process *in vivo* and may be produced in large quantities under ischemic and hypoxic conditions (4). The primary biological activity of adenosine is to increase the heart rate through the inhibition of the vagus nerve at low concentrations. At high concentrations, adenosine leads to bradycardia and atrioventricular block by inhibiting the cardiac conduction system. In addition, adenosine exhibits anti-adrenergic activities and causes contraction of glomerular arterial smooth muscle, while causing the expansion of systemic arterial smooth muscles. Therefore, adenosine demonstrates a regulatory role in coronary blood flow. The effect of adenosine on the degree of coronary artery ectasia has a positive correlation with the intravascular concentration of adenosine. The expansion of systemic arterial smooth muscles by adenosine may be triggered by the activation of adenosine 2 (A2) receptors located on the coronary vessel wall.

Adenosine stress echocardiography (ASE) refers to cardiac ultrasound detection, based on the action of adenosine on coronary artery dilatation. During the cardiac ultrasound examination, the injection of adenosine leads to the abnormal distribution of regional blood flow between the coronal-dominated lesion area and the normal area, due to the coronary ‘steal’ effect, resulting in possible myocardial ischemia. At present, in the diagnosis of coronary heart diseases, ASE is predominantly used for the patients who are unable to complete the exercise stress test. In addition, ASE is used to detect any viable myocardium remaining subsequent to an old myocardial infarction and the coronal blood flow reserve. However, the value of ASE for predicting the prognosis of patients with AMI has rarely been evaluated.

With the development of medical technology, percutaneous coronary intervention (PCI) has become one of the main treatments for coronary heart disease, and has been widely used in clinical practice (5). There is a requirement for a tool that is able to evaluate the efficacy of coronary heart disease treatment and to assess the conditions affecting the prognosis. ASE, as a non-invasive diagnostic technique, is a relatively safe and reliable diagnostic tool, and its value in clinical practice is gradually being recognized.

An evaluation standard for determining the efficacy of PCI is the restoration of blood flow to the ischemic myocardium by perfusion. Previous studies have shown that adenosine stress imaging demonstrates a high predictive value following PCI (6,7). In certain countries, ASE is also utilized for the preoperative diagnosis and assessment of the coronary artery. In the present study, the use of ASE in the examination of patients with AMI following PCI was evaluated, and the predictive value of ASE following PCI was investigated.

## Patients and methods

### Clinical data

Seventy-nine patients with AMI, treated with PCI in the Beijing Shijitan Hospital Affiliated to Capital Medical University (Beijing, China) from January 2007 to January 2010, were selected as subjects for the present study. The patients included 48 males and 31 females with an average age of 55.40±8.77 years. The diagnosis of AMI was in accordance with the criteria formulated by the Chinese Society of Cardiology Branch in 2001 and defined in the ‘AMI diagnostic criteria for the diagnosis of acute myocardial infarction and treatment guidelines’ (http://www.docin.com/p-521071293.html. Accessed February 27, 2009). AMI was initially diagnosed at 3–10 days according to the AMI diagnostic criteria, and the data in the present study were in good accordance with the criteria. The results of cardiac ultrasound showed motion abnormalities of the ventricular wall. The 79 patients all underwent PCI. Prior written and informed consent was obtained from every patient and the study was approved by the ethics review board of Capital Medical University.

### Low-dose adenosine stress echocardiography (LDASE)

Patients were assessed with by LDASE one week subsequent to the PCI. The patients ceased from taking medication such as calcium antagonists, β-blockers and nitrates prior to the LDASE examination. During the examination, the patients were supine, with upper extremity venous connections following disinfection to intravenous pumps. Doses of 80, 100 and 110 μg/kg/min adenosine were respectively injected via the intravenous pump (cat. no. H20030319; Shenyang Guangda Pharmaceutical Co., Ltd., Shenyang, China), and heart rate and blood pressure monitoring and two-dimensional ultrasound echocardiography (2DE) were performed. In order to complete the ASE, the injection dosage progressed from low to high, with each dose comprising a continuous infusion for 3 min. A GE Vivid 7 color Doppler ultrasound diagnostic system (GE Vingmed, Horten, Norway) was applied as a monitoring instrument, with the frequency of the probe adjusted to 2.5/3.5 MHz. Patients with wall motion were analyzed by two sonographers, according to the standards recommended by the American Heart Association.

### Test grouping, detection indicators and follow-up

The patients were divided into two groups according to the improvements in the LV systolic function following the LDASE. Subsequent to the injection of adenosine, the patients with ≥5% increases in the LV ejection fraction (LVEF) levels were assigned to the improved LVEF group, while the patients with <5% increases in LVEF levels were assigned to the unimproved LVEF group. All patients were classified by Killip classification and received a 2DE examination and an assessment of plasma B-type natriuretic peptide (BNP) levels.

### Enzyme-linked immunosorbent assay (ELISA)

An ELISA kit (R&D Systems, Inc., Minneapolis, MN, USA) was used and the detection was performed in accordance with the kit instructions. A 2DE examination, including LVEF and LV end-diastolic diameter (LVDd), was also performed. There were 42 patients in the improved LVEF group, including 22 males and 16 females, with an average age of 56.08±9.40 years. Thirty-seven patients were in the unimproved LVEF group, comprising 22 males and 15 females, with an average age of 54.98±8.37 years. There were no statistically significant differences between the two groups of patients with regard to average age, LVEF, plasma BNP levels and admission Killip classification. All patients were followed-up for a period of 24 months and the Killip classification, plasma BNP levels and 2DE parameters of the patients were monitored. Furthermore, certain parameters of the two groups of patients, such as the incidence of major adverse cardiovascular events (MACEs), new or worsening heart failure and non-fatal myocardial infarction were monitored.

### Multivariate unconditional logistic regression analysis

A range of risk factors associated with clinical prognosis were analyzed, including Killip classification, plasma BNP levels, LVEF, LVDd, the number of improved segments following adenosine stress, and LVEF and LVDd following adenosine stress testing.

### Statistical analysis

All data were analyzed with SPSS statistical software version 18.0 (SPSS, Inc., Chicago, IL, USA). The data are presented as the mean ± standard deviation and the differences between the groups were analyzed using a t-test. The count data are expressed as a percentage of the differences, with the differences between the groups analyzed using a χ^2^ test. Multivariate analysis was performed using an unconditional logistic regression analysis. P<0.05 was considered to indicate a statistically significant difference.

## Results

### Twenty-four-month follow-up investigation of the two groups of patients

To determine the value of ASE in the prognosis of AMI following PCI, a comparison of the two groups of patients within a 24-month follow-up period was performed. As shown in Table I, the incidence of MACEs in the improved and unimproved LVEF groups was 14.29 (6/42) and 43.24% (16/37), respectively. The difference in the incidence of MACEs between the two groups of patients was statistically significant (χ^2^=14.339, P=0.004). The incidence of new or worsening heart failure in the unimproved LVEF group was significantly higher compared with that in the improved LVEF group (37.84 versus 14.29%; χ^2^=23.170, P=0.000). The incidence of recurrent non-fatal myocardial infarction was also significantly higher in the unimproved LVEF group than in the improved LVEF group (0.00 versus 5.41%; χ^2^=20.512, P=0.000).

### Regression analysis of clinical prognostic variable factors of PCI between the two groups of patients

To investigate the risk factors for clinical prognosis between the two groups of patients, regression analysis of the clinical prognostic variable factors between the groups was performed. As shown in Table II, the occurrence of cardiovascular events following PCI in the 24-month follow-up investigation was expressed as a dependent variable. Logistic regression analysis of multivariate factors was performed using each candidate factor, in order to determine the independent risk factors for clinical prognosis. The independent risk factors were identified to be LVEF increases of <5% and segment improvements of ≤3 under adenosine stress conditions.

## Discussion

This study aimed to evaluate the clinical prognostic values of patients with AMI using ASE, following recanalization. Patients with AMI who underwent PCI were examined by ASE one week subsequent to the PCI surgery. It was observed that, in comparison with the improved LVEF group, the incidences of new or worsening heart failure and recurrent non-fatal myocardial infarction in the unimproved LVEF group were significantly higher. This was determined by monitoring the incidence of MACEs. Logistic regression analysis of clinical prognostic factors was also conducted in the study. The analysis showed that the independent risk factors were increases in LVEF of <5% and segment improvements of ≤3 under adenosine stress conditions. The study demonstrated that the use of ASE for patients with AMI following PCI is an effective method for evaluating LV function subsequent to reperfusion therapy. In addition, the results indicate that ASE may be used for the early prediction of cardiovascular adverse events following interventional treatment for AMI, thus facilitating the use of the therapy for clinical applications.

Following AMI, myocardial cells suffering from hypoxic-ischemic injury adapt to the adverse hypertrophy, the reduced number of myocardial cells and extracellular matrix accumulation and fibrosis, which leads to LV remodeling. LV remodeling results in increases in myocardial mass and ventricular volume and ventricular shape changes. Although the early phase may be characterized by an expansion of the infarct zone itself, late remodeling involves the whole ventricle, resulting in a time-dependent dilatation and alteration of ventricular shape. A major determinant of ventricular remodeling is known to be the infarct size (8). A further important predictor of remodeling has been shown to be the patency of the infarct-related artery, even though late patency of the infarct-related artery has been shown to limit LV remodeling, which indicates the benefit of improved myocardial reperfusion for the areas remaining viable (9,10). This is a secondary pathophysiological reaction of lesion repair and ventricular decompensation. LV remodeling is closely associated with heart failure, cardiac rupture, ventricular aneurysm, severe arrhythmia and death. Medical therapy has been shown to prevent, at least partially, LV remodeling if it is initiated soon after AMI (11). Thus, ventricular remodeling has been used as a reliable surrogate for long-term outcome, and the prevention of post-infarct ventricular remodeling has been considered as a primary target for treatment (11–14). The early detection of patients with a high risk of LV remodeling may aid in the identification of patients who are likely to benefit most from aggressive medical therapy. Different modalities have been used to predict LV remodeling, with the majority based on an assessment of infarct size. This approach is based on the known correlation between infarct size and the extent of LV remodeling. Serial measurements of serum creatine kinase and the extent of ST-segment elevation have been applied. Although the two methods are easily available, they exhibit considerable limitations in the precise assessment of infarct size and the prediction of LV remodeling (15,16). The more accurate analytical methods regarding LV remodeling predominantly include magnetic resonance imaging and radionuclide imaging (17). However, those techniques are confined in their clinical applicability due to limited availability.

Further studies with large numbers of patients are required to validate the conclusions of the present study. The recovery of LV function is closely associated with microcirculatory perfusion and myocardial viability. ASE does not reflect myocardial perfusion and microvascular circulation at the tissue level. However, it has been revealed that myocardial contrast echocardiography (MCE) is able to accurately predict the extent of microvascular damage and LV remodeling in AMI and ischemic heart disease (18). This may be the direction of future research.

In conclusion, LDASE has a high predictive value for LV remodeling in patients with AMI following revascularization. The technique may be used to identify the risk of significant LV remodeling and MACEs, which may be of use in clinical decision-making and risk stratification.

## Figures and Tables

**Table I:** Comparison of prognosis with 24-month follow-up between the two groups of patients.

Group	Cases (n)	Incidence of MACEs [% (n)]	Incidence of new or worsening heart failure [% (n)]	Incidence of non-fatal myocardial infarction [% (n)]	Incidence of cardiac death [% (n)]
Improved LVEF	42	14.29 (6)	14.29 (6)	0.00 (0)	0.00 (0)
Unimproved LVEF	37	43.24 (16)	37.84 (14)	5.41 (2)	0.00 (0)

MACEs, major adverse cardiac events; LVEF, left ventricular ejection fraction. Improved LVEF, ≥5% increase in LVEF levels; unimproved LVEF, <5% increase in LVEF levels.

**Table II:** Logistic regression analysis of the risk factors for clinical prognosis between the two groups of patients.

Relevant factors	Regression coefficient	Standard deviation	Wald	OR	P-value
Killip classification	0.921	0.104	18.334	2.423	0.193
Plasma BNP	1.533	0.314	15.451	4.632	0.200
LVEF	0.088	0.664	12.209	2.115	0.147
LVDd	0.503	0.603	3.145	2.560	0.311
LVEF increase ≤5% after ASE	1.214	0.482	15.156	2.669	0.002
LVEF increase >5% after ASE	2.336	0.757	2.806	2.359	0.227
Improved segments ≤3 after ASE	0.554	0.504	4.521	2.418	0.000
Improved segments >3 after ASE	0.803	0.111	10.323	2.117	0.118

OR, odds ratio; BNP, B-type natriuretic peptide; LVEF, left ventricular ejection fraction; LVDd, left ventricular end-diastolic diameter; ASE, adenosine stress echocardiography.

## References

[1] Huang X, Zhang L, Yuan Y (2007). Emergency interventional therapy of acute myocardial infarction and revascularization time relationship. Southern Medical University Science Bulletin.

[2] Masci PG, Ganame J, Francone M (2011). Relationship between location and size of myocardial infarction and their reciprocal influences on post-infarction left ventricular remodelling. Eur Heart J.

[3] Marfella R, Di Filippo C, Portoghese M (2009). Tight glycemic control reduces heart inflammation and remodeling during acute myocardial infarction in hyperglycemic patients. J Am Coll Cardiol.

[4] Simoni J, Simoni G, Wesson DE, Feola M (2012). ATP-adenosine-glutathione cross-linked hemoglobin as clinically useful oxygen carrier. Curr Drug Discov Technol.

[5] Zhao YL, Ao HS (2011). Research progress of myocardial ischemia-reperfusion injury. Chinese Circulation J.

[6] Ma D, Yao H, Liu H (2010). Clinical value of adenosine ^99m^Tc-MIBI myocardial perfusion single photon emission computed tomography in percutaneous coronary intervention. Chinese Circulation J.

[7] Liu Y, Li Z, Yang Y, Wang Y (2008). Assessment of myocardial viability in patients with myocardial infarction using strain rate imaging combined with low dose adenosine stress echocardiography. Chinese Journal of Ultrasound in Medicine.

[8] Sanchis J, Bodí V, Insa LD (1999). Predictors of early and late ventricular remodeling after acute myocardial infarction. Clin Cardiol.

[9] Lamas GA, Flaker GC, Mitchell G, The Survival and Ventricular Enlargement Investigators (1995). Effect of infarct artery patency on prognosis after acute myocardial infarction. Circulation.

[10] Jeremy RW, Hackworthy RA, Bautovich G (1987). Infarct artery perfusion and changes in left ventricular volume in the month after acute myocardial infarction. J Am Coll Cardiol.

[11] Sharpe N, Smith H, Murphy J (1991). Early prevention of left ventricular dysfunction after myocardial infarction with angiotensin-converting-enzyme inhibition. Lancet.

[12] Touchstone DA, Beller GA, Nygaard TW (1989). Effects of successful intravenous reperfusion therapy on regional myocardial function and geometry in humans: a tomographic assessment using two-dimensional echocardiography. J Am Coll Cardiol.

[13] Ito H, Maruyama A, Iwakura K (1996). Clinical implications of the ‘no reflow’ phenomenon. A predictor of complications and left ventricular remodeling in reperfused anterior wall myocardial infarction. Circulation.

[14] Pfeffer MA, Braunwald E, Moyé LA, The SAVE Investigators (1992). Effect of captopril on mortality and morbidity in patients with left ventricular dysfunction after myocardial infarction. Results of the survival and ventricular enlargement trial. N Engl J Med.

[15] Nixdorff U, Erbel R, Rupprecht HJ (1996). Sum of ST-segment elevations on admission electrocardiograms in acute myocardial infarction predicts left ventricular dilation. Am J Cardiol.

[16] Bosimini E, Giannuzzi P, Temporelli PL (2000). Electrocardiographic evolutionary changes and left ventricular remodeling after acute myocardial infarction: results of the GISSI-3 Echo substudy. J Am Coll Cardiol.

[17] Wu KC, Zerhouni EA, Judd RM (1998). Prognostic significance of microvascular obstruction by magnetic resonance imaging in patients with acute myocardial infarction. Circulation.

[18] Galiuto L, Garramone B, Scarà A, AMICI Investigators (2008). The extent of microvascular damage during myocardial contrast echocardiography is superior to other known indexes of post-infarct reperfusion in predicting left ventricular remodeling: results of the multicenter AMICI study. J Am Coll Cardiol.

